# Molecular Identification of Isolated Fungi from Unopened Containers of Greek Yogurt by DNA Sequencing of Internal Transcribed Spacer Region

**DOI:** 10.3390/pathogens3030499

**Published:** 2014-06-25

**Authors:** Irshad M. Sulaiman, Emily Jacobs, Steven Simpson, Khalil Kerdahi

**Affiliations:** Southeast Regional Laboratory, U.S. Food and Drug Administration, 60, Eighth Street NE, Atlanta, GA 30309, USA; E-Mails: emily.jacobs@fda.hhs.gov (E.J.); steven.simpson@fda.hhs.gov (S.S.); khalil.kerdahi@fda.hhs.gov (K.K.)

**Keywords:** nucleotide sequencing, rapid identification, foodborne disease

## Abstract

In our previous study, we described the development of an internal transcribed spacer (ITS)1 sequencing method, and used this protocol in species-identification of isolated fungi collected from the manufacturing areas of a compounding company known to have caused the multistate fungal meningitis outbreak in the United States. In this follow-up study, we have analyzed the unopened vials of Greek yogurt from the recalled batch to determine the possible cause of microbial contamination in the product. A total of 15 unopened vials of Greek yogurt belonging to the recalled batch were examined for the detection of fungi in these samples known to cause foodborne illness following conventional microbiological protocols. Fungi were isolated from all of the 15 Greek yogurt samples analyzed. The isolated fungi were genetically typed by DNA sequencing of PCR-amplified ITS1 region of rRNA gene. Analysis of data confirmed all of the isolated fungal isolates from the Greek yogurt to be *Rhizomucor variabilis*. The generated ITS1 sequences matched 100% with the published sequences available in GenBank. In addition, these yogurt samples were also tested for the presence of five types of bacteria (*Salmonella*, *Listeria*, *Staphylococcus*, *Bacillus* and *Escherichia coli*) causing foodborne disease in humans, and found negative for all of them.

## 1. Introduction

The primary mission of U.S. Food and Drug Administration is to enforce the Food, Drug and Cosmetic Act and regulate food, drug and cosmetics. This act prohibits the distribution of adulterated food which includes those products that are defective, unsafe, and filthy or produced under unsanitary conditions. To accomplish this mission, FDA uses the presence of human-pathogenic microorganisms that include bacteria, virus, parasites and fungi in these commodities as one of the regulatory action criteria [1].

Approximately 300 individuals reported developing gastrointestinal symptoms after consuming Greek yogurt in several states of United States including Arizona, Delaware, New York, Maryland, Ohio, Pennsylvania, California, Indiana and Florida. The consumers also stated bloating and swelling of some of the products. The potentially affected products were manufactured and distributed nationwide from one of its facility, and the consumers received the products through retail and club stores. The company voluntarily recalled more than half-a-dozen yogurt varieties with expiration dates September 11 to October 07 of 2013, and stopped distribution of the product. Later, the yogurt maker made an announcement that *Mucor circinelloides* was the potential microorganism that triggered the recall of some of its Greek yogurt, and *Salmonella* or *E*. *coli* were not associated with the foodborne illnesses.

Although the kingdom fungi are estimated to have approximately 1.5 million to 5 million species, its taxonomy is still constantly changing with only 5% of the species being formally classified. However, to this point approximately 100,000 fungal species have been described [2]. Nevertheless, DNA sequencing has been widely effective in identification of fungi both at inter- and intra- species level. The internal transcribed spacer (ITS) region of rRNA gene has been found to be the most suitable genetic marker for fungal typing, and also considered as a universal DNA barcode to ascertain the fungal taxonomy [3,4,5,6].

Fungi are recognized as one of the main contaminants of dairy products that include yogurt and sour milk. However, these microorganisms can also cause spoilage in a wide range of processed, preserved and refrigerated food products. Conversely, a number of fungi (some species of yeast) are considered as a significant group of microorganisms in food industry for their positive contributions in fermentation of bread, alcoholic beverages and cheese [7,8]. Thus far, for the species-specific identification of spoilage fungi, a number of molecular methods have been developed. Of these, DNA sequencing of PCR amplified ITS products, has resulted best diagnostic results. The presence of fungi is also considered as environmental microbiological indicator [9].

In our previous study, we described the development of a molecular method based on ITS1 sequencing that can identify fungi at the species level. Using this DNA sequencing protocol, we successfully typed the isolated fungi from the manufacturing premises of a compounding company known to have caused the multistate fungal meningitis outbreak [10].

In this study, we have examined the unopened vials of Greek yogurt from the recalled batch to determine the microbial contamination in the product. The samples were tested for the detection of human-pathogenic fungi and five types of bacteria (*Salmonella*, *Listeria*, *Staphylococcus*, *Bacillus* and *Escherichia coli*) causing foodborne illness in humans. These samples were found negative for all five types of bacteria tested. However, all of the samples analyzed were found positive for the presence of fungi. The recovered fungal isolates were genetically characterized by our recently described method based on DNA sequencing of PCR-amplified ITS1 products [10]. Using ITS1 region as the genetic marker, a species-specific identification was achieved. The results further supported that the ITS1 region is a reliable benchmark for rapid detection and differentiation of human-pathogenic fungi of public health importance.

## 2. Results and Discussion

A total of fifteen unopened vial of Greek yogurt were analyzed initially by conventional microbiologic protocol for the detection of fungi (yeast and molds) and five human-pathogenic bacteria (*Salmonella*, *Listeria*, *Staphylococcus*, *Bacillus*, and *E. coli*) known to cause foodborne illness following FDA’s BAM protocol [11]. All Greek yogurt samples examined in the study were found negative for the presence of all five types of bacteria tested. However, the analysis confirmed the unopened vials of yogurt samples to be 100% positive for fungi for all 15 sealed yogurt samples tested. The fungal isolates were recovered from both liquid (Sabouraud Dextrose Broth) and semi-solid (Malt Extract Agar) enrichment media used. The Sabouraud Dextrose broth is commonly used for the isolation of fungi from food, pharmaceutical and cosmetic products and in Environmental Monitoring and Sterility Testing. The high dextrose concentration and acidic pH of this broth permit selectivity of fungi as the low pH not only favors fungal growth but also inhibits the growth of contaminating bacteria [11,12]. The Malt Extract contains polysaccharides that provides energy source and makes the medium acidic, and the peptone provides the nitrogen source [11].

For all recovered fungal isolates from Greek yogurt, genomic DNA was extracted and a two-step nested PCR was performed at least twice to amplify the ITS1 region of the rRNA gene. Using the ITS1 amplified products for all isolated fungi, a bi-directional DNA sequencing was completed with high quality bases (>98% HQ–100% HQ).

Analysis of the generated ITS1 nucleotide sequences confirmed species-identification to all isolated fungi from the unopened containers of Greek yogurt. All of the ITS1 nucleotide sequences obtained in this study matched 100% with the published sequence of *Rhizomucor variabilis* (GenBank Accession No. JF904893). No intra-specific genetic variation was noticed among the recovered fungi from the 15 closed containers of Greek yogurt (Table 1).

Sanger sequencing is a method of DNA sequencing of a gene or genome (complete or partial). This tool has been most extensively used over two decades including for the completion of “Human Genome Project” to get the first human genome, and is considered as the gold standard for genetic analysis. Sequence characterization of human-pathogenic microorganisms has been widely used for their rapid detection from sporadic cases, surveillance and outbreak investigations. This molecular tool has not only provided species-specific identification but also helped in conducting epidemiologic surveys to determine the transmission dynamics of emerging infectious diseases of public health importance [13].

Of the various conserved or regulatory gene used as a genetic marker, the rRNA has been proven to be most successful in developing rapid diagnostic methods for the identification of human-pathogenic microorganisms as well as their vectors [14,15,16]. As this locus exist in all organisms often with several copies in tandem repeats evolving at slower evolutionary rate, it is characterized as the prime target to establish phylogenetic relationship [17,18]. Of various rRNA regions characterized, the internal transcribed spacer region has been found to be a reliable marker to understand the genetic polymorphism at inter—as well as intra—species level. A surveillance study was conducted over a span of 13 months in four counties of eastern Maryland, involving 465 fur-bearing mammals for human-pathogenic microsporidia, *Enterocytozoon* spp. The ITS sequence characterization described 15 new genotypes of *E*. *bieneusi* in wildlife for the first time [19]. The ITS sequencing confirmed the genotype distribution of *E*. *bieneusi* in HIV-infected patients in Lima, Peru by analyzing stool samples from 89 microsporidia-positive patients, and the analysis identified 11 distinct genotypes of *E*. *bieneusi* (Peru-1 to Peru-11). Of these, six were new genotypes, and the remaining five genotypes had ITS sequences identical to those previously reported in humans, cats, pigs, and wild mammals [14]. The ITS sequencing also identified 32 PCR positive *E*. *bieneusi* samples belonging to 338 fecal samples collected from cattle farms in Florida, Maryland, Michigan, New York, North Carolina, Pennsylvania, Virginia, and Portugal. Results of this study indicated that microsporidiosis was prevalent in cattle and there was an extensive genetic diversity of *E*. *bieneusi* isolates found in them [18].

**Table 1:** Identification of fungal isolates recovered from Greek yogurt by DNA sequencing of internal transcribed spacer (ITS)1 region of rRNA gene.

Sample Analyzed	Species Identified	Sequence Type	% Similarity (bp)	Reference
GYSW-1	*Rhizomucor variabilis*	rRNA	100 (291)	JF904893, This study
GYSW-2	*Rhizomucor variabilis*	rRNA	100 (291)	JF904893, This study
GYSW-3	*Rhizomucor variabilis*	rRNA	100 (291)	JF904893, This study
GYSW-4	*Rhizomucor variabilis*	rRNA	100 (291)	JF904893, This study
GYSW-5	*Rhizomucor variabilis*	rRNA	100 (291)	JF904893, This study
GYSW-6	*Rhizomucor variabilis*	rRNA	100 (288)	JF904893, This study
GYSW-7	*Rhizomucor variabilis*	rRNA	100 (288)	JF904893, This study
GYSE-1	*Rhizomucor variabilis*	rRNA	100 (288)	JF904893, This study
GYSE-2	*Rhizomucor variabilis*	rRNA	100 (288)	JF904893, This study
GYSE-3	*Rhizomucor variabilis*	rRNA	100 (288)	JF904893, This study
GYSE-4	*Rhizomucor variabilis*	rRNA	100 (288)	JF904893, This study
GYSE-5	*Rhizomucor variabilis*	rRNA	100 (291)	JF904893, This study
GYSE-6	*Rhizomucor variabilis*	rRNA	100 (291)	JF904893, This study
GYSE-7	*Rhizomucor variabilis*	rRNA	100 (291)	JF904893,This study
GYSE-8	*Rhizomucor variabilis*	rRNA	100 (291)	JF904893, This study

Representative sequences generated in this study have been deposited in GenBank, with accession numbers KJ162161 to KJ162175.

Furthermore, the ITS region has also been recognized as a suitable genetic marker for species-specific identification in fungi [3,4,5,20,21,22,23,24,25]. In recent years, the ITS1 region has been widely sequenced for genetic typing of human-pathogenic clinical isolates of fungi causing allergies, respiratory illness, cutaneous infection and life-threatening meningitis. The ITS1 sequence analysis identified 40 human-pathogenic yeast species belonging to 106 clinical strains [3]. The sequencing of the ITS region also identified 201 strains of 44 species of human-pathogenic molds [26]. Targeting the ITS1 region, a multiplex PCR detection method was developed to detect and differentiate five of the *Rhizopus* species causing meningitis in human [23]. The ITS sequencing was also successful in an outbreak investigation of intestinal mucormycosis infection caused by *Rhizopus* [27].

Since the ITS region is now being considered as formal fungal barcodes, recommendation has been to create a separate public reference data set for ITS fungal sequences [28]. Attempts have also been made in establishing guidelines for the authenticity and reliability of newly generated fungal ITS sequences [29]. In a separate study, attempts were made to describe how the commonly used fungal ITS primers are hampered by different types of biases that include length, taxonomic and primer mismatch [30].Furthermore, ITS-based sequencing has been an effective tool for environmental testing focusing on potential health effects of fungal exposure. In a study, the indoor environments was tested by comparing ITS sequence profile of the recovered fungal flora from the indoor dust collected from a moisture damaged nonindustrial building and compared with an undamaged one [24]. More recently, we sequenced the ITS1 region and characterized a total of 26 environmental swab samples collected from various locations of the manufacturing premises of a Compounding Center known to have caused the 2012 multistate fungal meningitis outbreak in United States. The ITS1 typing confirmed the presence of 15 distinct fungal species belonging to 12 different genera among the swabs analyzed [10].

Fungi are commonly associated with the spoilage and bio-deterioration of a large variety of food. A number of DNA-based molecular methods have been developed and used for their detection in food. Reverse transcription (RT)-PCR and real-time RT-PCR assays were developed targeting actin locus for rapid detection and quantification of viable yeasts and molds contaminating yogurt and pasteurized food products [31]. A PCR assay was developed using the conserved region of 18S rRNA gene for the detection of yeast in dairy products [32]. For the detection of viable yeast in yogurt samples, this region was used as the genetic marker in developing a PCR and RT-PCR assays as well [33,34]. Although, the firm voluntary recalled more than half-a-dozen yogurt varieties with the specific code and expiration dates, and acknowledged *Mucor circinelloides* to be the pathogen that triggered the recall of some of their products. However, when these samples from the same batch known to have caused the gastrointestinal disease in individuals who had consumed this product were sequence characterized at ITS1 region, DNA sequencing confirmed the isolated fungi in all 15 unopened vials of Greek yogurt analyzed to be *Rhizomucor variabilis* (this study). The data analysis also confirmed absence of genetic variations among the generated ITS1 sequences of the isolated fungi, and these sequences matched completely with the available sequence of *Rhizomucor variabilis* in the public domain.

## 3. Experimental Section

### 3.1. Isolation of Salmonella, Listeria, Staphylococcus, Bacillus and E. coli from Greek Yogurt

A total of fifteen Greek yogurt samples were analyzed for the detection of five pathogenic bacteria (*Salmonella*, *Listeria*, *Staphylococcus*, *Bacillus* and *E*. *coli*), known to cause the foodborne illness in humans by following FDA’s Bacteriological Analytical Manual (BAM) and American Association of Analytical Chemists (AOAC) protocols [11,35]. In all cases, the positive and negative controls were also analyzed simultaneously.

The *Salmonella* analysis was performed using the Enzyme-Linked Fluorescent Assay (ELFA) Screening Method [11,35]. The test portion was prepared by compositing 25 g from each of the 15 units of product for a total of 375 g. A total of 3375 g of lactose (BD Difco™, Sparks, MD, USA) pre-enrichment broth was added and incubated for 24 h at 35 °C. The overnight pre-enrichment was then inoculated into two selective enrichment broths, 0.1 mL into 10 mL Rappaport-Vassiliadis (RV) medium and 1.0 mL into 10 mL Tetrathionate (TT) broth, and incubated for 24 h at 42 °C. Later, 1 mL from each of the RV and TT broth was inoculated into 10 mL M broth (BD Difco™), incubated for 6 h at 42 °C, and tested to detect *Salmonella* using the ELFA based VIDAS system and *Salmonella* SLM assay kit (bioMérieux, Marcy-l’Etoile, France), in the Greek yogurt sample analyzed. The analysis was terminated, if the results were negative.

For the analysis of *Listeria monocytogenes*, the test portion from 10 units was weighed into two composites, and each composite consisted of 50 g from each of the 5 units for a total of 250 g following the BAM and AOAC protocols [11,35]. Then an equal amount of pre-enrichment broth (demi-Fraser broth, BD Difco™) was added to each composite, and homogenized for 2 min. From each sample, 50 g of homogenate was removed and an additional 200 g of demi-Fraser broth was added to make a final ratio of 1:9 products to broth, and incubated for 25 h at 30 °C. After incubation, 1 mL portion of the pre-enrichment was then inoculated into 10 mL Fraser broth (BD Difco™), incubated 25 h at 30 °C, and tested for the detection of *Listeria* species using the ELFA based VIDAS system and *Listeria* LIS assay kit (bioMérieux). The analysis was terminated, if the results were negative.

The *Staphylococcus aureus* analysis was performed using the most probable number method following the BAM protocol [11]. Individual units of products were first diluted to 10^−1^ by removing 50 g and adding 450 mL of Butterfield’s phosphate buffer (BPO_4_) (Fisher Scientific, Fair Lawn, NJ, USA), and homogenized. The 10^−1^ dilution was further diluted to 10^−2^, 10^−3^, and 10^−4^ by serially diluting the 10 mL of previous dilution to 90 mL BPO_4_. Then 1 mL of each serial dilution (10^−1^, 10^−2^, 10^−3^, and 10^−4^) was transferred in triplicate into 10 mL Trypticase Soy (TSB) broth (Acumedia, Lansing, MI, USA) containing 10% NaCl (Fisher Scientific, Fair Lawn, NJ, USA) and 1% Sodium Pyruvate (Fisher Scientific), and incubated at 35 °C for 48 h. If any turbidity was noticed, it was streaked on to a Baird-Parker (BP) agar plate (Acumedia, Lansing, MI, USA), with Egg Yolk Tellurite (BD Difco™, Franklin Lakes, NJ, USA), and incubated at 35 °C for 48 h. After incubation, the BP plates were observed for bacterial growth. If no typical colonies of *S*. *aureus* observed, the analysis was terminated.

The *Bacillus* analysis was also performed by using the most probable number protocol [11]. The product was analyzed as individual units, and diluted to 10^−1^ dilution by weighing 50 g of product and adding 450 mL BPO_4_ buffer (Fisher Scientific), and homogenized. The 10^−1^ dilution was further diluted to 10^−2^ and 10^−3^ by serially diluting 10 mL of previous dilution to 90 mL BPO_4_. Then 1 mL of all serial dilutions (10^−1^, 10^−2^, and 10^−3^) were transferred in triplicate, into 10 mL TSB containing Polymyxin B (Sigma, St. Louis, MO, USA), and incubated at 30 °C for 48 h. Any turbid tubes were then sub-cultured to Mannitol-Egg Yolk-Polymyxin (MYP) (Oxoid, Hampshire, UK) with polymyxin B (Sigma), Egg yolk emulsion (BD Difco™) agar plates, and incubated at 30 °C for 48 h. If *B*. *cereus* specific pink colonies were not noticed, the analysis was terminated.

For the presence of the Total Coliform and *Escherichia coli* in the Greek yogurt sample, the AOAC Total Coliform and *Escherichia coli* in All Foods, Substrate Supporting Disc Method was used [36]. Individual product units were diluted 10^−1^ by homogenizing 50 g of product with 450 mL of BPO_4_ buffer (Fisher Scientific). The 10^−1^ dilution was further diluted to 10^−2^, 10^−3^, and 10^−4^ by serially diluting 10 mL of previous dilution to 90 mL BPO_4_. Then 1 mL of all of the above serial dilutions (including 10^−1^, 10^−2^, 10^−3^, and 10^−4^) were transferred in triplicate into 10 mL Lauryl Tryptose (LST) broth (BD Difco™). Afterwards, one ColiComplete Substrate Supporting Disc (BioControl Systems, Inc., Bellevue, WA, USA) containing 5-bromo-4-chloro-3-indolyl-b-d-galactopyranoside (X-Gal) and 4-methylumbelliferyl-b-d-glucuronide (MUG) was added to each LST tube. The LST tubes were then incubated at 35 °C for 30 h, and observed for blue color and fluorescence under long wave UV light (366 nm). In the absence of UV fluorescence and blue color the product was considered negative for the presence of coliforms and *E*. *coli*.

### 3.2. Isolation of Fungi from the Unopened Containers of Greek Yogurt

To recover fungi from the Greek yogurt samples, a loop full of each yogurt sample was inoculated into a sterile tube containing 100 mL of sterile Sabouraud Dextrose (BD Difco™) broth, and incubated at 25 °C for 5 days. After completion of 5 days of incubation, the broth was streaked in duplicate to Malt Extract (BD Difco™) agar plates, and incubated at 25 °C for an additional 7 days. In addition, all the samples were also directly streaked on these plates, and incubated at 25 °C for 7 days. These plates were read and the growth was recorded after 24 h interval every day for the next 7 days. Fungi observed on any of the agar plates were examined by staining and microscopy. The sterile media were tested as negative control. Furthermore, *Candida albicans* (ATCC #10231) and *Aspergillus brasilensis* (ATCC #16404) cultures were carried through the analysis to serve as positive culture controls. The positive and negative controls were analyzed concurrently.

### 3.3. DNA Extraction

For DNA extraction, approximately 100 mg of fungal specimen was homogenized in a clean and autoclaved mortar and pestle, if desired liquid nitrogen was used. The homogenate was purified by RNeasy Plant Mini Kit DNA, following manufacturer’s protocol and recommendations for the purification of total genomic DNA from fungi (QIAGEN, Valencia, CA, USA). The concentration of purified DNA samples was measured at 260-nm absorbance using a NanoDrop-1000 spectrophotometer (NanoDrop Technology, Rockland, DE, USA), and stored at −20 °C until used.

### 3.4. PCR Amplification

The fragments of ITS region of rRNA gene was amplified by a previously described forward (5'-TCC GTA GGT GAA CCT GCG G-3') and reverse (5'-GCT GCG TTC TTC ATC GAT GC-3') primer set [3,9] was used, with modified PCR conditions [20]. A total of 50 µL PCR reaction consisted of 25 µL of HotStarTaq Master Mix (QIAGEN), and 25 µL of a solution containing 200 nM of each primer, 1.5 mM of additional MgCl_2_ (Promega, Madison, WI, USA) and the template DNA (50 ng) diluted in PCR grade water. The QIAGEN HotStarTaq Master Mix is a premixed solution containing HotStarTaq DNA Polymerase, PCR Buffer, and dNTPs with a final concentration of 1.5 mM MgCl_2_ and 200 µM each dNTP. The PCR reactions were run for 35 cycles (each cycle is 94 °C for 45 s, 55 °C for 45 s, and 72 °C for 60 s) in a GeneAmp PCR 9700 thermocycler (Applied Biosystems, Foster City, CA, USA), with an initial hot start (94 °C for 15 min) and a final extension (72 °C for 10 min). The PCR products were analyzed by agarose gel electrophoresis, and visualized after ethidium bromide staining.

### 3.5. Nucleotide Sequencing

The PCR products were enzymatically cleaned before cycle sequencing by an addition of 3 µL of the ExoSAP‐IT (USB Corporation, Cleveland, OH, USA) to 5 µL of each amplified PCR product. The mixture was incubated at 37 °C for 20 min followed by 80 °C for 15 min on a GeneAmp PCR 9700 thermocycler (Applied Biosystems). The purified PCR products were sequenced using AB Big-Dye 3.1 dye chemistry (Applied Biosystems). A total of 20 μL sequencing reactions contained 2 μL of cleaned PCR product, 1 μL of BigDye Terminator v3.1 Ready Reaction Mix, 2 μL of 5× Sequencing Buffer, 1.6 pmol of Forward or Reverse sequencing primer and PCR grade water. The sequencing reactions were performed for 25 cycles (each cycle is 96 °C for 30 s, 50 °C for 15 s, and 60 °C for 4 min) and hold at 4 °C in a GeneAmp PCR 9700 thermocycler (Applied Biosystems). The sequencing reactions were then cleaned with the Performa^®^ DTR Gel Filtration Cartridges (Edge Bio, Gaithersburg, MD, USA) following manufacturer’s protocol (Edge Bio), and sequenced using the AB 3500 XL automated DNA sequencers (Applied Biosystems).

### 3.6. Data Analysis

The accuracy of nucleotide sequence was achieved by performing two-directional DNA sequencing, and by sequencing of a new PCR-amplified product if necessary. Multiple alignments of the nucleotide sequences were completed by BioEdit and Geneious programs with manual adjustments. Nucleotide sequences of the ITS region of rRNA gene of recovered fungal species produced in the study were deposited in GenBank database under accession number KJ162161 to KJ162175.

## 4. Conclusions

In conclusion, our results confirmed that the recovered fungal species from all of the 15 unopened Greek yogurt containers to be *Rhizomucor variabilis*. Our findings suggest that the presence of this human-pathogenic microorganism may be the reason for the recent gastrointestinal disease reported in individuals who consumed the Greek yogurt of this particular batch. The results suggest that the ITS1 region is a reliable benchmark for rapid detection and differentiation of human-pathogenic fungi and molds. This molecular detection tool will help in achieving the mission of our agency by notably analyzing food contaminated with molds of public health importance.
